# Sophoridine exerts tumor-suppressive activities via promoting ESRRG-mediated β-catenin degradation in gastric cancer

**DOI:** 10.1186/s12885-020-07067-x

**Published:** 2020-06-22

**Authors:** Zhiyang Peng, Qing Guan, Jianfei Luo, Wenhong Deng, Jiasheng Liu, Ruicheng Yan, Weixing Wang

**Affiliations:** 1 Department of Gastrointestinal Surgery in East Hospital, Renmin Hospital of Wuhan University, No. 6 Gaoxin Road, East Lake High-tech Development Zone District, Wuhan, 430205 P.R. China; 2 Department of Laboratory Medicine, Xiangyang Central Hospital, Affiliated Hospital of Hubei University of Arts and Science, Xiangyang, 441021 P.R. China

**Keywords:** Gastric cancer, Sophoridine, ESRRG, β-Catenin

## Abstract

**Background:**

As a natural alkaloid product isolated from *Sophora alopecuroides. L*, Sophoridine reshapes gastric cancer immune microenvironment via inhibiting chemotaxis and M2 polarization of tumor-associated macrophages (TAMs). However, the exact effects and underlying mechanism of Sophoridine on gastric cancer cells remains poorly known.

**Methods:**

The potential anti-tumor effects of Sophoridine on gastric cancer cell lines, including AGS and SGC7901 cells, were detected by CCK-8, EDU and colony forming assay, immunofluorescence, transwell assay, and flow cytometry. Molecular mechanisms of Sophoridine were investigated by siRNA transfection, nuclear/cytoplasmic extraction and western blot. The synergistic effects of Sophoridine with cisplatin on gastric cancer cells were further investigated in in vitro functional studies.

**Results:**

Sophoridine exhibited potent tumor-suppressive activities in gastric cancer cells, including inhibition of proliferation, colony formulation, migration and invasion, as well as induction of apoptosis. In addition, we further showed that Sophoridine induced G2/M cell cycle arrest via inhibiting double-stranded DNA breaks repair and enhanced the efficacy of cisplatin in gastric cancer cells. Molecular studies further revealed that Sophoridine promoted β-catenin degradation by enhancing Estrogen-related receptor gamma (ESRRG) expression, but not depended on ubiquitination-proteasome pathway, either TRIM33-mediated (GSK3β-independent) or altered GSK3β activity, and thus exerted potent tumor-suppressive activities.

**Conclusion:**

Sophoridine depends on targeting ESRRG/β-catenin pathway to exert tumor-suppressive activities in gastric cancer cells and enhances the anti-tumor effect of cisplatin. Our study provided the promising preclinical anti-tumor evidence for the potential application of Sophoridine against gastric cancer.

## Background

Pharmaceutical molecules screened from medicinal plants and herbs provide the 60% of currently used anti-tumor agents [1]. In this context, numerous alkaloids, such as camptothecin, vincristine, homoharringtonine and vinflunine, have been approved for clinical use as agents for the treatment of hematological and lymphatic neoplasms [2]. Homoharringtonine, a clinically approved drug for leukemia, sensitized cancer cells to TRAIL-induced necroptosis through the RIPK1/RIPK3/MLKL pathway [3]. Vinflunine has been approved as a second-line therapeutic drug for metastatic and advanced urothelial cancer after failure of platin-containing therapy, and also showed potential therapeutic benefit for EGFR2-positive metastatic breast cancer along with trastuzumab in a phase II clinical trial [4–6]. As a potent inhibitor of P-gp efflux pump to reverse multidrug resistance, tetrandrine (CBT-01®) has demonstrated positive results in a phase I clinical trial in combination with paclitaxel, which warranted conducting it’s phase II/III trial [7]. For now, over 21,000 different alkaloids belong to different subclasses, like indole and isoquinoline alkaloids, have been identified in more than 300 plant families [2]. Specifically, these alkaloids within a particular structural class have been shown to exhibit differential cellular and molecular mechanisms and showing varied cytotoxicity against various cancer. Thus, a better understanding of the anti-tumor molecular mechanisms of alkaloids is emergently needed for their clinical application.

Sophoridine is an active quinolizidine alkaloid isolated from leaves of Leguminous plant *Sophora alopecuroides. L*. Accumulating evidence demonstrated that Sophoridine displays remarkable pharmacological effects in inflammatory diseases [8], infectious diseases [9] and cancers [10]. In particular, Sophoridine and its derivatives have drawn more and more attention owing to their potent anti-tumor effects in different tumor cell lines and animal models [11–13]. The underlying anti-tumor mechanisms of Sophoridine including increase of intracellular ROS levels, activation of the phosphorylation of ERK and JNK, induction of S phase arrest in pancreatic cancer cells [14]; inhibition of growth and invasion in human colorectal cancer cells via directly binding to MAPKAPK2 and inactivates its phosphorylation [15]; inhibition of ubiquitin-proteasome pathway in human glioma cells [16]. In gastric cancer, one of the most common and deadly neoplasms, evidence have shown that Sophoridine reshapes gastric cancer immune microenvironment via inhibiting chemotaxis and M2 polarization of tumor-associated macrophages (TAMs), and thus leading to the increased proliferation and cytotoxic function of CD8^+^ T cells [17]. However, the direct effects and underlying mechanisms of Sophoridine on gastric cancer cells still remain unclear.

Here, we demonstrated that Sophoridine exerts potent tumor-suppressive activities directly on gastric cancer cells, including inhibition of proliferation, colony formulation, migration and invasion, as well as induction of apoptosis of gastric cancer cells. In addition, we further showed that Sophoridine induces the G2/M phase and EMT process arrest in gastric cancer cells. Molecular studies revealed that Sophoridine depends on Estrogen-related receptor gamma (ESRRG) to perform tumor-suppressive activities and which promotes the degradation of β-catenin, but not ubiquitin-proteasome pathway. Thus, our study provided the promising preclinical anti-tumor evidence for the potential application of Sophoridine against gastric cancer.

## Methods

### Cell culture

Human normal gastric epithelial cell line (GES-1) and gastric cancer cell lines (AGS and SGC7901) were purchased from Cell Bank, Type Culture Collection Committee of Chinese Academy of Sciences (http://www.cellbank.org.cn/, CAMS, Shanghai, China). Cells were grown in Dulbecco’s modified essential medium or RPMI1640 supplemented with 10% fetal bovine serum (FBS), 100 U/mL penicillin and 100 μg/mL streptomycin at 37 °C in a humidified incubator with 5% CO_2_. Sophoridine was purchased from MedChemExpress (Shanghai, China) and dissolved in dimethyl sulfoxide (DMSO) to prepare a 10 mM stock solution for use.

### Cell viability assay

Cell viability in response to Sophoridine treatment was determined using CCK-8 assay (Beyotime, Shanghai, China). In brief, cells seeded in flat bottom 96 well plates (5 × 10^3^ cells/ 100 μL) were either treated with Sophoridine at indicated concentrations or treated with indicated drugs for 24 h. Subsequently, CCK-8 solution (10 μL/well) was added an0064 followed by 4 h of incubation. The absorbance was detected by Spectra-Max 190 microplate reader (Molecular Devices) at 450 nm. The percentages of survival cells were measured based on the absorbance of DMSO-treated cells.

### EdU assay

Gastric cancer cells with or without transfection were seeded in 96-well plates at a density of 5 × 10^3^ cells/well and then treated with Sophoridine (3 μM) for 24 h. Subsequently, the cells were incubated with a final condition of 10 μM EdU (Beyotime) for 2 h at 37 °C. Next, supernatant was discarded, and cells were fixed with 4% paraformaldehyde for 30 min. The cells were then treated with 0.5% Triton X-100 for 10 min and rinsed with PBS three times. Thereafter, the cells were exposed to 100 μL of click reaction cocktail (Azide 647 to label EdU, Beyotime) for 30 min and then incubated with 5 μg/mL of Hoechst 33342 to stain the cell nuclei for 30 min. Images were captured using Olympus IX73 microscope. The percentage of EdU-positive cells in each filed (six random fields were counted in each assay) was defined as the proliferation rate. All the experiments were performed in triplicate.

### Colony formation assay

AGS and SGC7901 cells (1 × 10^3^) were seeded into 6 well plates. After 24 h, cells were treated with Sophoridine (3 μM) at the indicated concentrations for 24 h. Cells were then cultured in fresh medium for another week. Colonies fixed with methanol and stained with 0.05% crystal violet for 30 min. Photographs were acquired and colonies containing more than 50 cells were counted. All the experiments were performed in triplicate.

### Immunofluorescence assay

Cells were seeded in 24 well plates and treated with Sophoridine at indicated concentrations for 24 h. The cells were washed in cold PBS and then fixed with 4% paraformaldehyde. Subsequently, cells were blocked with 1% BSA containing 1% goat serum for 30 min. After incubation with mouse monoclonal antibodies to E-cadherin or N-cadherin (Abcam, Shanghai, China) overnight at 4 °C, cells were exposed to Alexa Fluor® 647 labelled goat polyclonal secondary antibody (Abcam) for 1 h at room temperature, and then stained with DAPI. Cells were observed by using Olympus IX73 microscope.

### Cell transfection

ESRRG and its non-targeted control (siNC) siRNAs were synthesized from RiboBio (Guangzhou, China). Transfection were performed with Lipofectamine 3000 (Invitrogen) following the manufacturer’s protocol. Selective silencing performance was identified by western blot.

### Preparation of nuclear and cytoplasmic fractions

Nuclear and cytoplasmic extractions were performed using an NE-PER™ Nuclear Cytoplasmic Extraction Reagent kit (Thermofisher, Shanghai, China) according to the manufacturer’s protocol. In brief, the treated cells (2 × 10^6^) were washed twice with ice-cold PBS and centrifuged at 500 g for 3 min, and then, cell pellet was re-suspended in 200 μL of cytoplasmic extraction reagent I (CER I) by vertexing. Cell suspension was subsequently incubated on ice for 10 min followed by the addition of 11 μL of a second cytoplasmic extraction reagent II (CER II), vertexing for 5 s, incubation on ice for 1 min, and centrifuged at 16000 g for 5 min. Supernatant was then transferred to a pre-chilled tube (cytoplasmic fraction). The insoluble pellet fraction contained crude nuclei was then resuspended in 100 μL of ice-cold nuclear extraction reagent (NER) by vertexing for 15 s every 10 min over a total period of 40 min, and then centrifuged at 16000 g for 10 min. The resulting supernatant contained the nuclear nuclear fraction was collected for use.

### Western blot

Cells were seeded in six-well plates and treated with indicated conditions. Total proteins were extracted from cells with RIPA buffer (Beyotime) containing proteinase inhibitor at the indicated time points and then determined concentrations by the BCA reagent kit (Beyotime). Equal amounts of proteins (30 μg) were separated by sodium dodecyl sulfate -polyacrylamide gel electrophoresis and followed by transferring to polyvinylidene difluoride (PVDF) membrane (Millipore, USA). The membranes were then blocked with 5% non-fat dry milk in TBST buffer for 1 h, and then probed with primary antibodies against HSP27, BIRC3, p53, Bid, pGSK3β, GSK3β, E-cadherin, N-cadherin, Vimentin, snail, TRIM33, ESRRG (Abcam), caspase 3, total and phospho-β-catenin, γH2AX, RAD51, α-tubulin (CST), p21, BCL2, HDAC1 (Santa Cruz) at 4 °C overnight. Next, the membranes were washed in TBST buffer and incubated with anti-mouse or rabbit horseradish peroxidase-conjugated secondary antibodies. Target proteins were visualized by using the enhanced chemiluminescence system (Millipore) and quantified by ImageJ software (Version 6.0, Media Cybernetics, Inc.).

### Flow cytometry

For cell cycle analysis, cells were trypsinzed, washed in PBS, fixed in 70% ice-cold ethanol and stored at − 20 °C overnight. Samples were then re-suspended in PBS and stained with 50 μg/mL propidium iodide (PI) solution containing 0.2% Triton X-100 and 100 μg/mL DNase-free RNase A for analysis. For apoptosis analysis, cells were harvested and stained using FITC-Annexin V/PI apoptosis detection Kit (BD Biosciences) according to the manufacturer’s instructions. For E-cadherin and N-cadherin expression, cells were trypsinized and washed in cold PBS. Subsequently, cells were blocked with 1% BSA containing 1% goat serum for 15 min and then incubated with primary antibodies to E-cadherin or N-cadherin for 20 min. Next, cells were exposed to Alexa Fluor® 647 labelled goat polyclonal secondary antibody (Abcam) for 1 h at room temperature. Cells were analyzed by using FACSCalibur (Becton Dickinson). Data analysis was performed using FlowJo version 7.6.1 software (TreeStar).

### Transwell assay

AGS and SGC7901 cells treated with indicated conditions were resuspended in serum-free RPMI-1640 medium, and 1 × 10^5^ cells were seeded into the upper 24-well chambers (8- μm pore size, Corning Costar). RPMI-1640 medium containing 20% FBS was added to the lower chambers. After 24 h, cells remaining on the upper surface of the membrane were removed with a cotton swab, and the cells that had migrated/invaded into another side of the membrane were fixed with methanol for 15 min. And then, cells were stained with 0.05% crystal violet for 30 min and photographed under Olympus IX73 microscope. The number of migration cells in each filed (six random fields were counted in each assay) was counted from three independent experiments.

### Statistical analysis

Results were expressed as mean ± SD and analyzed by using the Graphpad Prism V.5.00 software (GraphPad Software, CA, USA). Unpaired *t*-test or one-way ANOVA followed by Neuman-Keuls post-hoc test was used to determine the significance of the difference between groups. A *P* value less than 0.05 was considered statistically significant.

## Results

### Sophoridine inhibits proliferation and colony formulation in gastric cancer cells

**Fig. 1:**
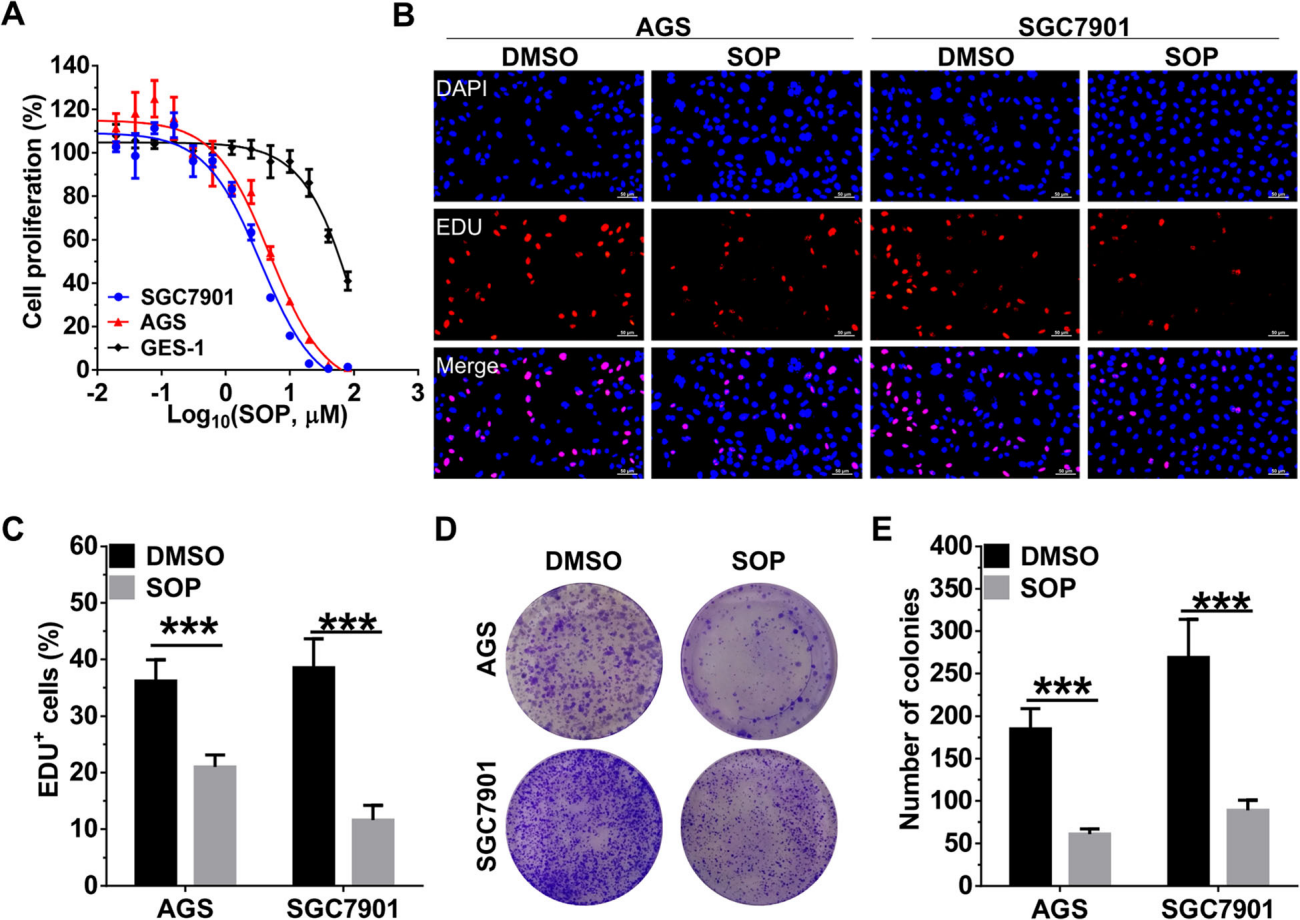
SOP inhibits proliferation and colony formulation in gastric cancer cells. **a** Human gastric epithelial cells (GES-1) and gastric cancer cell lines AGS, SGC7901 were treated with SOP in indicated concentrations for 24 h. Cytotoxicity was assessed with a CCK-8 assay, and IC50 values were calculated by Graphpad software. **b** AGS and SGC7901 cells were treated with or without 3 μM SOP for 24 h and then EdU assay was used to evaluate cell viability. **c** Statistical analysis of the EdU-positive cell ratio in AGS and SGC7901 cells. **d** AGS and SGC7901 cells were treated with 3 μM SOP and the clones were visualized by crystal violet staining. **e** Statistical analysis of colony numbers in AGS and SGC7901 cells. The results are representatives of at least 3 independent experiments. Data were presented as mean ± SD. ***P < 0.0001. Abbreviation: SOP, Sophoridine

As a monomeric alkaloid extracted from *sophora alopecuroides L*, sophoridine exhibited potent anti-tumor effects on human liver, pancreatic, gallbladder, colon and prostate cancer cells [14]. To further clarified the anti-tumor effects of Sophoridine on gastric cancer cells, we firstly measured the IC50 values of sophoridine on gastric cancer AGS and SGC7901 cell lines and normal gastric epithelial cell line GES-1 by the CCK-8 assay. SGC7901 and AGS cells were more sensitive to the cytotoxic effects of Sophoridine with IC50 values of 3.52 μM and 3.91, respectively. GES-1 cells exhibited less sensitivity to Sophoridine with IC50 values of 51.40 μM, indicating that Sophoridine selectively kills gastric cancer cells (Fig. 1a). Next, we further performed EdU and colony formation assay to confirm the cytotoxic effect of Sophoridine on gastric cancer cells. As shown in Fig. 1b and c, Sophoridine significantly inhibited the proliferation of AGS and SGC7901 cells, which was reflected by the decrease of EdU-labelled S phase cells. In colony formation assay, Sophoridine treatment also led to a significant inhibition of monolayer cell growth and colony formation (Fig. 1d and e).

### Sophoridine induces apoptosis and G2/M phase arrest in gastric cancer cells

**Fig. 2:**
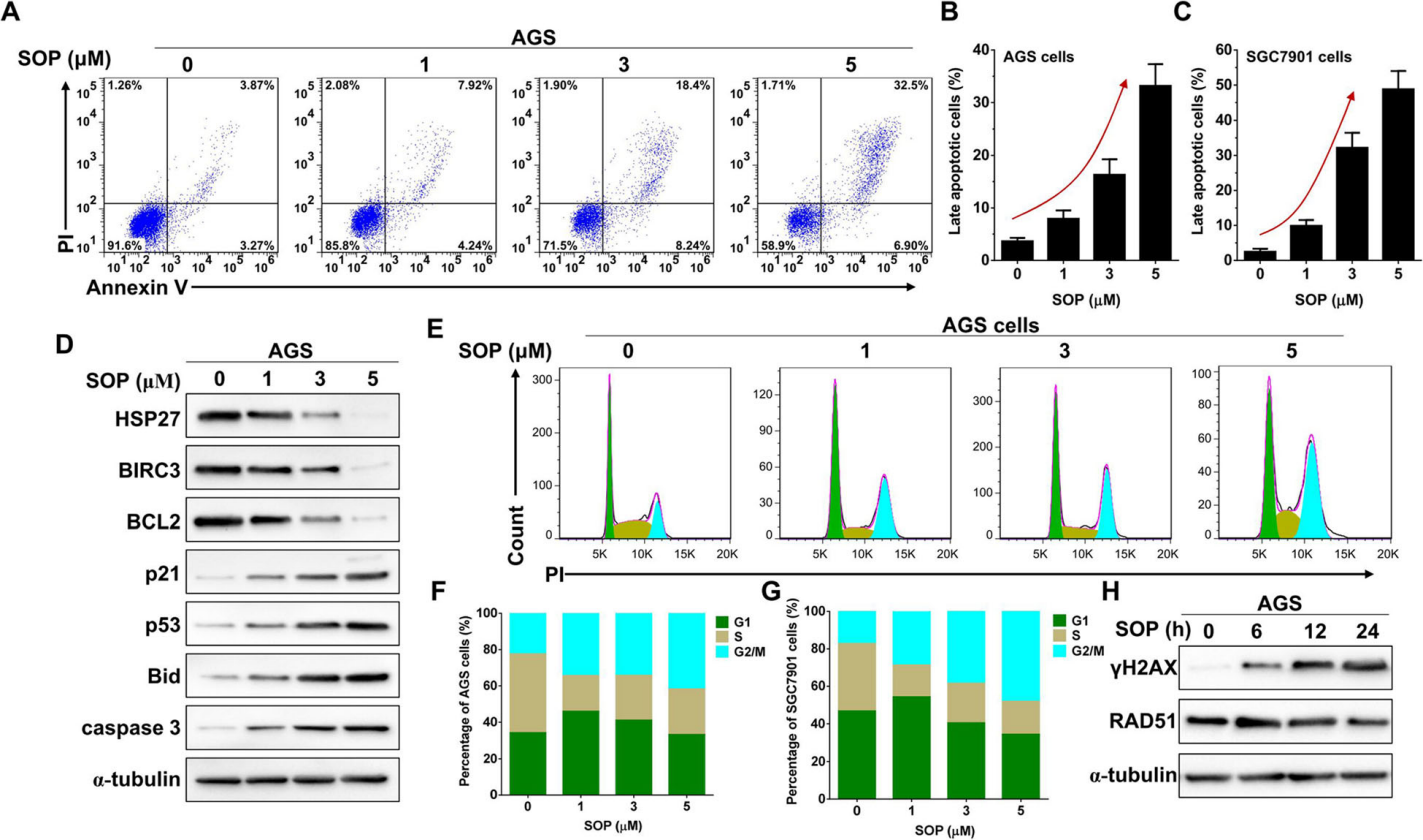
SOP induces apoptosis and G2/M phase arrest in gastric cancer cells. **a** AGS cells were treated with indicated concentrations of SOP for 24 h, Annexin V-FITC/PI stain and flow cytometry analysis were performed to assess apoptosis. **b** Statistical analysis of the Annexin V^+^PI^+^ cell ratio in AGS cells. **c** Statistical analysis of the Annexin V^+^PI^+^ cell ratio in SGC7901 cells. **d** Western blot analysis of the expression of apoptosis related proteins in AGS cells treated with indicated concentrations of SOP for 24 h. Full-length blots/gels are presented in Supplementary Figure S4. **e** AGS cells were treated with indicated concentrations of SOP for 24 h, PI stain and flow cytometry analysis were performed to assess cell cycle distribution. **f** Statistical analysis of cell cycle phase ratio in AGS cells. **g** Statistical analysis of cell cycle phase ratio in SGC7901 cells treated with indicated concentrations of SOP for 24 h. **h** AGS cells were treated with or without 3 μM SOP for indicated hours, and then γH2AX and RAD51 expression were determined by western blot. Full-length blots/gels are presented in Supplementary Figure S4. The results are representatives of at least 3 independent experiments. Data were presented as mean ± SD. Abbreviation: SOP, Sophoridine

Next, the apoptotic effects of Sophoridine in gastric cancer cells were measured by Annexin V-FITC/PI double staining. In response to the dose increase of Sophoridine, percentage of late apoptotic cells (Annexin V^+^PI^+^ cells) in both AGS (Fig. 2a and b) and SGC7901 cell (Fig. 2c) lines were gradually increased. Specifically, compared with the DMSO control (0 μM), Sophoridine treatment increased late apoptotic population from 3.65% ± 0.64% (control) to 33.17% ± 4.14% (5 μM) in AGS cells and from 2.51% ± 0.83% (control) to 48.80% ± 5.19% (5 μM) in SGC7901 cells, respectively. Western blot analysis of AGS cells in response to Sophoridine treatment also showed that antiapoptotic proteins HSP27, BIRC3, and BCL2 levels were gradually decreased, whereas proapoptotic proteins, p21, p53, BID and caspase 3 levels were gradually increased (Fig. 2d). These results indicated that the activation of intrinsic pro-apoptotic pathways is induced by Sophoridine in gastric cancer cells.

In order to examine whether Sophoridine inhibited cell growth and induced cell apoptosis via inducing cell cycle disturbance, cell cycle distribution was analyzed and results showed that G2/M phase accumulation in AGS cells (Fig. 2e and f) and SGC7901 cells (Fig. 2g and Figure S1A) were gradually increased with the increase of Sophoridine dosage. Compared with the DMSO control (0 μM), Sophoridine treatment increased G2/M phase population from 22.49% (0 μM) to 41.76% (5 μM) in AGS cells and from 17.25% (control) to 48.09% (5 μM) in SGC7901 cells, respectively. To investigate whether Sophoridine inhibited DNA damage repair in G2/M phase, we analyzed the expression of phosphorylated H2AX (γH2AX, an early marker of DNA double-strand break) and RAD51 (recombinase involved in DNA homologous recombination repair) in different time points of Sophoridine (3 μM) treated AGS cells. Western blotting showed that the expression of γH2AX was gradually increased whereas RAD51 was decreased until 24 h post Sophoridine treatment (Fig. 2h). As γH2AX represent unrepaired DNA damage while RAD51 indicate homologous recombination repair progression, these results suggested that Sophoridine induces G2/M phase arrest in gastric cancer cells via inhibiting DNA damage repair.

### Sophoridine inhibits migration and invasion of gastric cancer cells

**Fig. 3:**
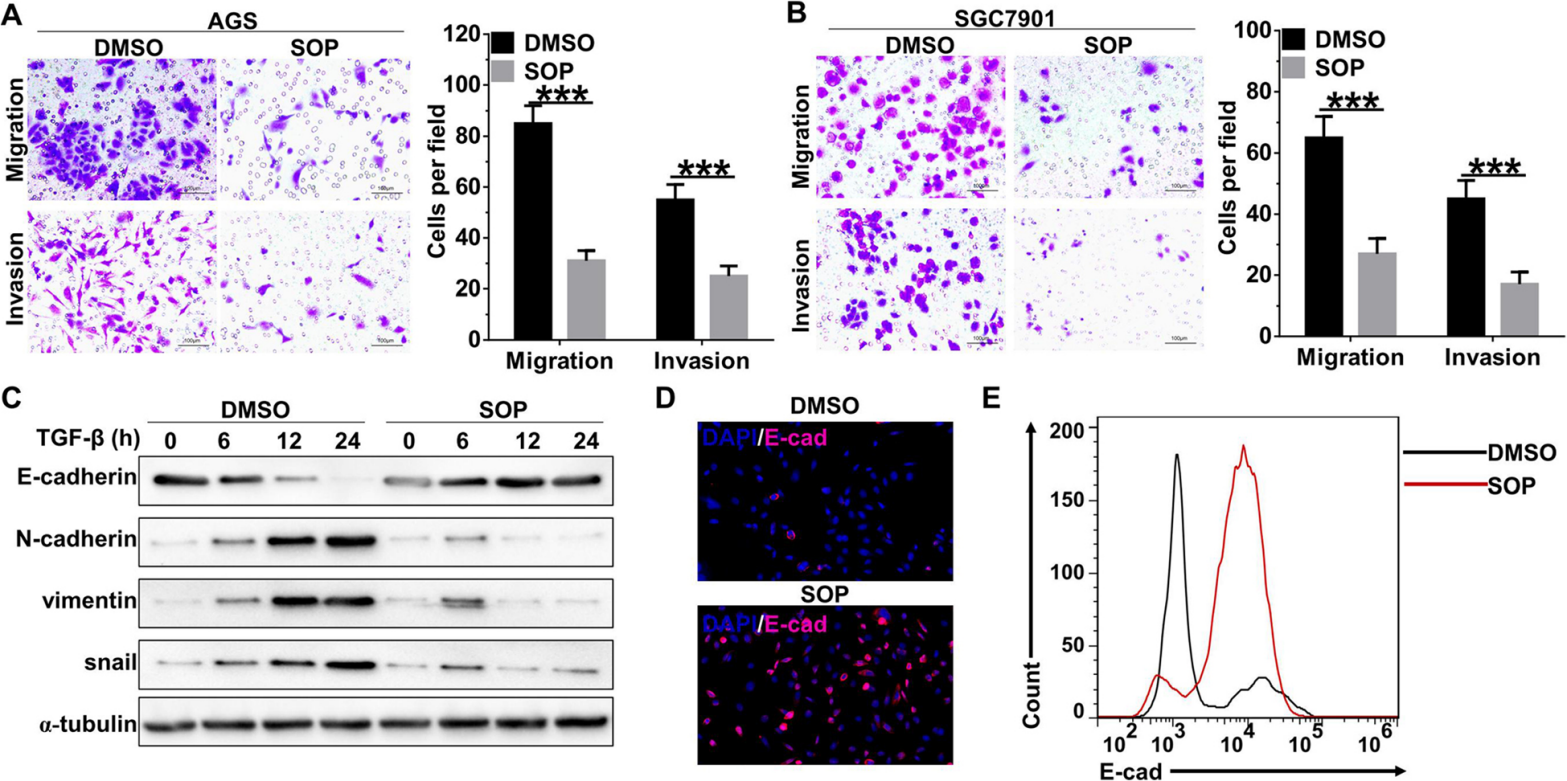
SOP inhibits migration and invasion of gastric cancer cells. **a** AGS cells were treated with or without 3 μM SOP for 24 h and then transwell assay was used to evaluate cellar migration and invasion. **b** SGC7901 cells were treated with or without 3 μM SOP for 24 h and then transwell assay was used to evaluate cellar migration and invasion. **c** AGS cells were treated with 5 ng/ml TGF-β alone or in combination with 3 μM SOP for indicated hours, the expression of EMT-related proteins was determined by western blot. Full-length blots/gels are presented in Supplementary Figure S5. E-cadherin expression in (**c**) was further determined by immunofluorescence (**d**) and flowcytometry (**e**) analysis. The results are representatives of at least 3 independent experiments. Data were presented as mean ± SD. ***P < 0.0001. Abbreviation: SOP, Sophoridine

Then, we further evaluated the effects of Sophoridine on migration and invasion of gastric cancer cells. Transwell assay revealed that migration and invasion of AGS cells (Fig. 3a) and SGC7901 cells (Fig. 3b) were significantly decreased with the treatment of Sophoridine. Furthermore, time course western blot analysis also showed that Sophoridine effectively blocked the epithelial-mesenchymal transition (EMT) process of AGS cells that induced by TGF-β treatment. Protein expression of the epithelial marker E-cadherin was significantly increased in TGF-β-treated AGS cells with the presence of Sophoridine, whereas mesenchymal markers, like N-cadherin, vimentin and snail were significantly decreased (Fig. 3c). These results were further confirmed by Immunofluorescence stain (Fig. 3d, Figure S1B) and flowcytometry (Fig. 3e, Figure S1C) analysis, as E-cadherin positive cells were remarkably increased while N-cadherin positive cells were decreased in the presence of Sophoridine in TGF-β-treated AGS cells. Collectively, these results indicated Sophoridine attenuates migration, invasion and EMT process of gastric cancer cells and EMT process.

### Sophoridine enhances β-catenin degradation by ESRRG in gastric cancer cells

**Fig. 4:**
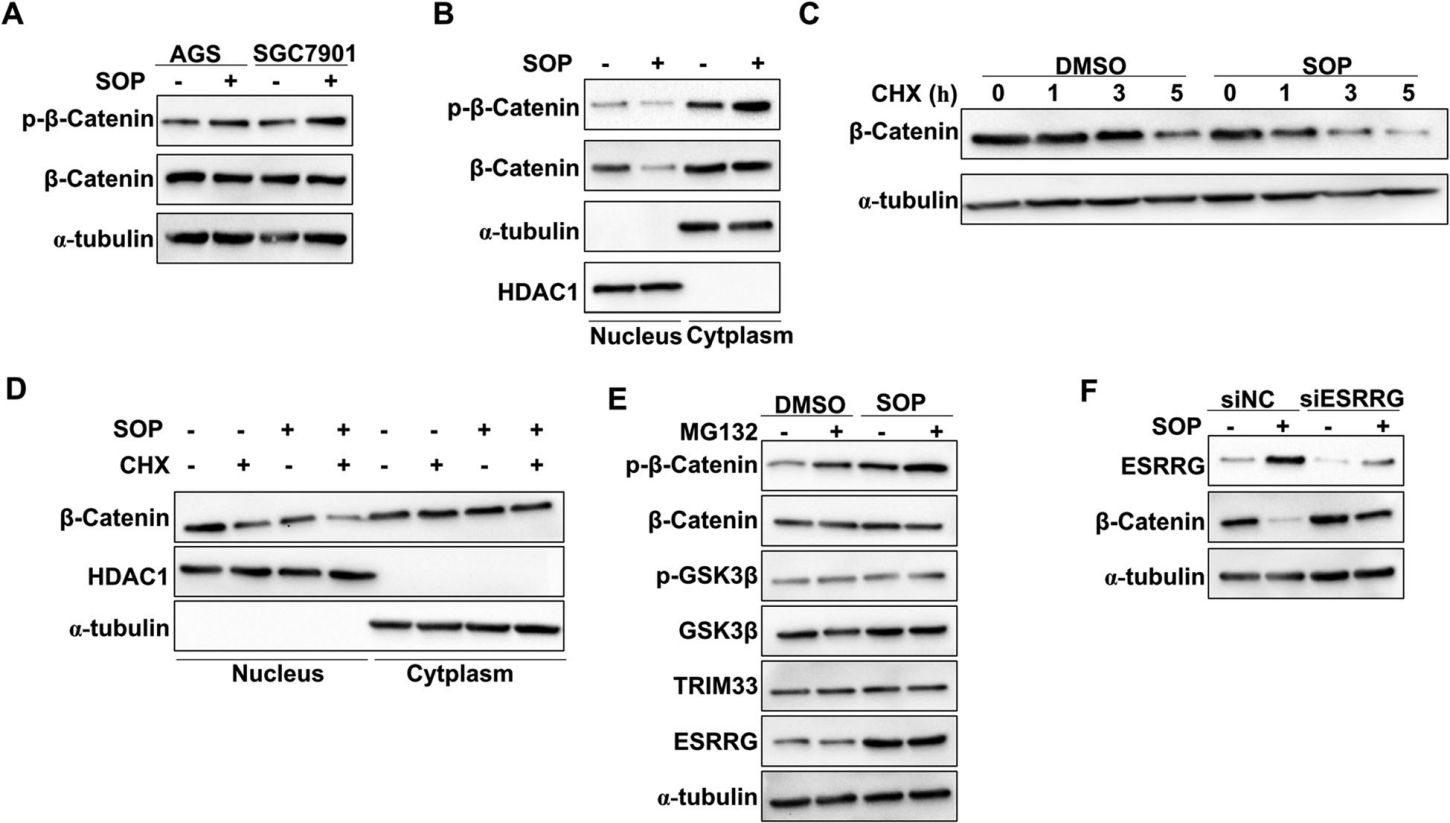
SOP enhances β-catenin degradation by ESRRG in gastric cancer cells. **a** AGS and SGC7901 cells were treated with or without 3 μM SOP for 24 h. phosphorylated β-catenin^(Ser33/37/Thr41)^ and β-catenin expression were determined by western blot. **b** phosphrylated β-catenin^(Ser33/37/Thr41)^ and β-catenin expression in cellular fractions of AGS cells (**a**) were detected. **c** AGS cells were treated with 1 μM CHX alone or in combination with 3 μM SOP for the indicated times and β-catenin expression were determined by western blot. (D) AGS cells were treated with 1 μM CHX alone or in combination with 3 μM SOP for 5 h, β-catenin expression in cellular fractions were detected by western blot. **e** AGS cells were treated with with 10 μM MG132 alone or in combination with 3 μM SOP for 24 h, expression of indicated proteins were determined by western blot. f AGS cells transfected with siRNA non-target control (siNC) or ESRRG siRNA were treated with or without 3 μM SOP for 24 h, ESRRG and β-catenin expression were determined by western blot. Full-length blots/gels are presented in Supplementary Figure S6 and band density of target proteins was quantified by ImageJ software (Version 6.0, Media Cybernetics, Inc.) as presented in Figure S2. The results are representatives of at least 3 independent experiments. Abbreviation: SOP, Sophoridine; CHX: cycloheximide

Activation of β-catenin is a frequent molecular event associated with the malignant transformation of gastric epithelial cells and also is an attractive therapeutic target being currently explored for cancer therapy [18]. In response to Sophoridine treatment, our results showed that the total β-catenin protein levels were almostly unaltered in AGS and SGC7901 cells, whereas the phosphorylated β-catenin level were increased (Fig. 4a, Figure S2A). Non-phosphorylated (activated) β-catenin increases the binding affinity of TCF4/LEF1 to target genes and is inactivated upon phosphorylation in canonical Wnt signaling. Activated β-catenin is localized in the nucleus to perform its function [19], we then next determined whether Sophoridine depended on cellular fraction to regulate β-catenin expression in AGS cells. As shown in Fig. 4b and Figure S2B, our results showed that ESRRG suppressed the expression of activated β-catenin in the nucleus but not in the cytoplasm. Furthermore, we also noticed that the phosphorylated β-catenin (inactive form) level in the cytoplasmic fraction was increased with the presence of Sophoridine (Fig. 4b, Figure S2B). Since active β-catenin is phosphorylated in the nucleus and then exported to cytoplasm for degradation, thus we hypothesized that Sophoridine could influence the stability of β-catenin. After pre-treated with the protein synthesis inhibitor cycloheximide (CHX), we measured the expression of active β-catenin in Sophoridine-treated AGS cells and found that β-catenin was more rapidly degraded in Sophoridine-treated cells (Fig. 4c, Figure S2C). In addition, the protein level of active β-catenin in Sophoridine-treated cells in response to CHX was decreased in the nuclear fraction but not in the cytoplasm (Fig. 4d, Figure S2D). GSK3β, TRIM33 (GSK3β-independent) and ESRRG are pivotal molecules that mediated the degradation of β-catenin [20]. To further confirm which molecule was the effector of Sophoridine, we then measured the expression of β-catenin and these three proteins in Sophoridine-treated AGS cells in the presence or absence of the proteasome inhibitor MG132. We found that β-catenin degradation by Sophoridine was not dependent on ubiquitination–proteasome pathway, either TRIM33-mediated (GSK3β-independent) or altered GSK3β activity, whereas the expression of ESRRG was increased in Sophoridine-treated cells (Fig. 4e, Figure S2E). Interestingly, we further found that ESRRG interference significantly blocked the downregulation of β-catenin expression induced by Sophoridine in AGS cells (Fig. 4f, Figure S2F). Taken together, these results demonstrated that Sophoridine decreases β-catenin stability by inducing ESRRG expression.

### Sophoridine depends on ESRRG to perform tumor-suppressive activities in gastric cancer cells

**Fig. 5:**
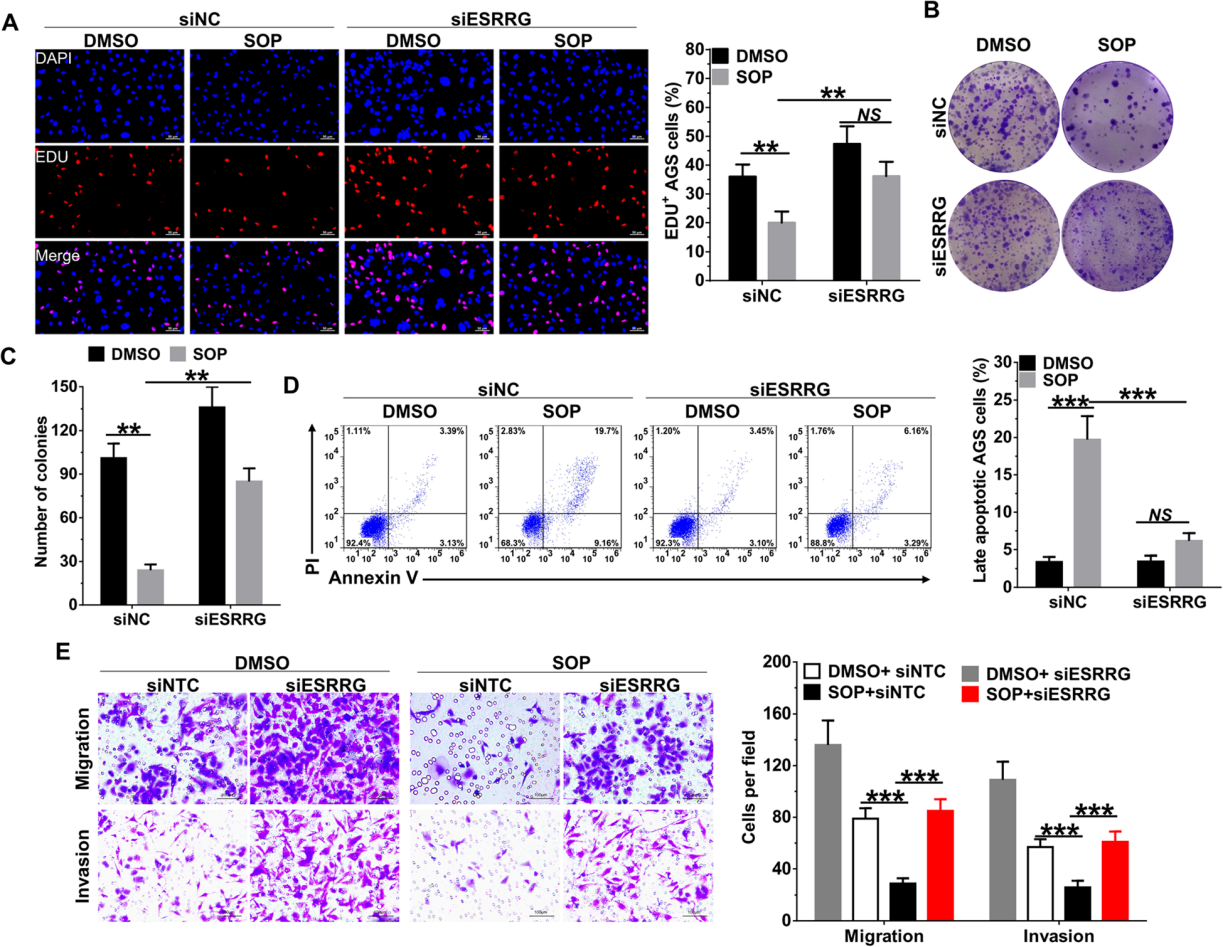
SOP depends on ESRRG to perform tumor-suppressive activities in gastric cancer cells. AGS cells transfected with siRNA non-target control (siNC) or ESRRG siRNA were treated with or without 3 μM SOP for 24 h. **a** EDU assay was used to evaluate cell viability; **b** the clones were visualized by crystal violet staining. **c** Statistical analysis of colony numbers in AGS cells. **d** Apoptotic AGS cells were determined by flowcytometry. **e** migration and invasion of AGS cells were determined by transwell assay. The results are representatives of at least 3 independent experiments. Data were presented as mean ± SD. **P < 0.01; ***P < 0.0001; *NS*, no significant difference. Abbreviation: SOP, Sophoridine

ESRRG is an important tumor suppressor in human breast, endometrial, prostate and gastric cancer [21, 22], we next investigated whether ESRRG is required for the anti-tumor activities of Sophoridine in gastric cancer cells. EdU and colony formation assay revealed that Sophoridine-mediated inhibition of proliferation was significantly decreased in ESRRG-interfered AGS (Fig. 5a-c) and SGC7901 cells (Figure S3A) when compared to siNC-transfected corresponding cells. We further found that ESRRG interference markedly blocked the proapoptotic (Fig. 5d, Figure S3B) and G2/M phase arrest (Figure S3C and S3D) effects of Sophoridine in AGS and SGC7901 cells. In addition, same as the effects on cell survival, ESRRG interference also reversed the effects of Sophoridine on migration and invasion of AGS cells (Fig. 5e) and SGC7901 cells (Figure S3E). Our data thus demonstrated that Sophoridine depends on ESRRG to induce β-catenin degradation and which contributes to its tumor suppressive properties in gastric cancer cells.

### Sophoridine enhances the effects of cisplatin in gastric cancer cells

**Fig. 6:**
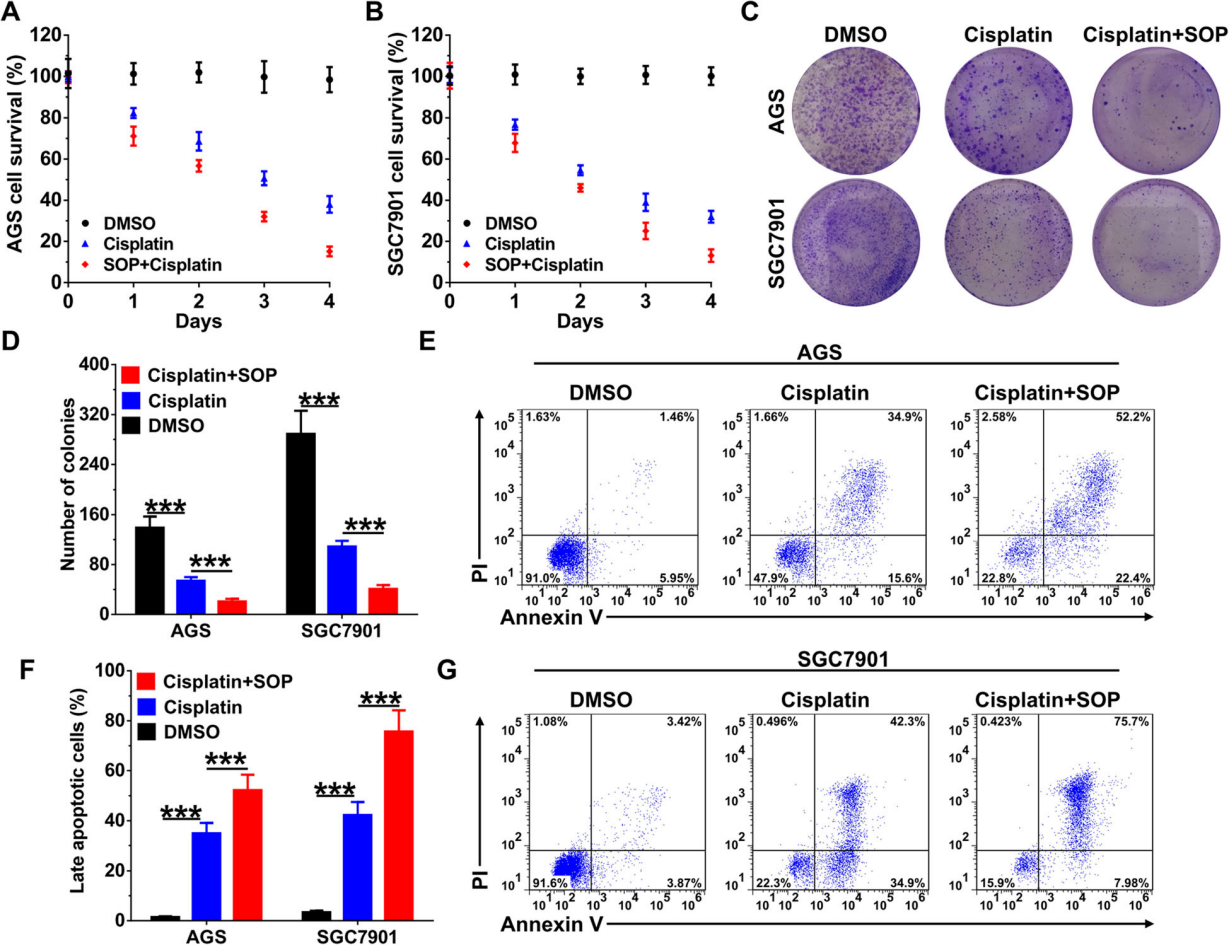
SOP enhances the effects of cisplatin in gastric cancer cells. **a** AGS cells and **b** SGC7901 cells were treated with 10 μM cisplatin alone or in combination with 3 μM SOP for indicated days and CCK-8 assay was used to determine cellular cytotoxicity. **c** colony formulation in AGS and SGC7901 cells treated with 10 μM cisplatin alone or in combination with 3 μM SOP were visualized by crystal violet staining. **d** Statistical analysis of colony numbers in AGS and SGC7901 cells. **e**-**g** Apoptosis in AGS and SGC7901 cells treated with 10 μM cisplatin alone or in combination with 3 μM SOP were determined by flowcytometry. Abbreviation: SOP, Sophoridine. The results are representatives of at least 3 independent experiments. Data were presented as mean ± SD. **P < 0.01; ***P < 0.0001; *NS*, no significant difference. Abbreviation: SOP, Sophoridine

Cisplatin is one of the common constituents of first-line treatment after surgery and a poor response to cisplatin is one of the causes of adverse effects in gastric cancer [23, 24]. Similar with the effects of Sophoridine on gastric cancer cells, cisplatin interferes with DNA replication, leading to G2/M cell cycle arrest and apoptosis. To investigate the translational potential of Sophoridine in gastric cancer, we compared the efficiency of Sophoridine and cisplatin combination with cisplatin alone in vitro. Results from CCK-8 assay revealed that combination of Sophoridine and cisplatin significantly inhibited more proliferation than single cisplatin alone in AGS (Fig. 6a) and SGC7901 (Fig. 6b) cells. Similar with the results from CCK-8 assay, combination of Sophoridine and cisplatin also significantly inhibited more colony formation than single cisplatin alone in AGS and SGC7901 (Fig. 6c and d) cells. In addition, we also observed combination of Sophoridine and cisplatin specifically induced more late stage apoptotic cells than cisplatin alone in AGS and SGC7901 cells (Fig. 6e-g). These results suggested that Sophoridine enhances the efficacy of cisplatin in gastric cancer cells.

## Discussion

Due to their diverse chemical structures and pharmacological effects, natural products and their derivatives are high-impact sources of new potential therapeutic anti-tumor agents [25]. As a natural product isolated from *Sophora alopecuroides. L*, Sophoridine exhibits potent anti-tumor effects against human colorectal carcinoma, medulloblastoma, glioma and pancreatic cancer cells [14, 15]. Furthermore, Sophoridine also polarized tumor-associated macrophages (TAMs) to M1-TAMs through TLR4/IRF3 axis and thus enhanced the cytotoxic function of CD8^+^ T cells in gastric cancer microenvironment in a recent report [17]. In the present study, we further demonstrated that Sophoridine directly inhibits cell growth and colony formation, invasion and migration, as well as induces cellular apoptosis of gastric cancer cells. Conventional chemotherapy is commonly recommended as a fundamental treatment for gastric cancer, however the clinical response rates to chemotherapeutic regimens are still no more than 50% and the clinical efficacy is usually modest, resulting in a median survival of 6 to 11 months [26]. Among chemotherapies, cisplatin is a platinum-based DNA-binding drug and results double-stranded DNA breaks in gastric cancer cells [27]. In the present study, Sophoridine inhibit double-stranded DNA breaks repair and enhances the efficacy of cisplatin in gastric cancer cells. Taking all these findings into consideration, Sophoridine may be a potent therapeutic candidate to improve gastric cancer outcomes due to has both chemo- and immunotherapeutic effects.

Sophoridine exhibits remarkable inhibitory effects on proliferation and induces apoptosis of pancreatic cancer cells via inducing MAPK signaling pathways (ERK and JNK)-triggered cell cycle arrest in G0/G1 phase or S phase [14]. In this study, we found that Sophoridine specifically decreases S phase but induces G2/M phase arrest in gastric cancer cells as revealed by EdU assay and flowcytometry analysis. In addition, we also noticed that Sophoridine suppresses TGF-β-induced EMT process, and the following migration and invasion depends on tumor-suppressor ESRRG, different from its effect in human colorectal cancer cells, in which Sophoridine inhibits cellular invasion via directly binding to MAPKAPK2 and inactivates its phosphorylation [15]. MAPKAPK2 and ESRRG are known to be acted as a downstream signaling protein of MAPK pathways, p38 and ERK respectively [15, 22, 28]. ERK is generally involved in proliferation and metastasis, while activation of JNK and p38 MAPKs are generally induced by oxidative stress and closely associated with apoptosis or inflammatory responses [29]. Thus, we could conclude that Sophoridine may mainly depend on MAPK pathways to exert its anti-tumor activities, but the exact activation status and corresponding role of individual MAPKs in response to Sophoridine treatment in different cancers still need to be investigated in future.

Sophoridine or its derivatives suppress activation of β-catenin in breast cancer, lung cancer and Hepatocellular Carcinoma [30] and hyperactivation of β-catenin plays important roles in promoting gastric cancer progression [18]. However, the potential mechanisms of Sophoridine-indcued inhibition on β-catenin activation is still unclear. In the present study, we found that Sophoridine promoted β-catenin degradation. To determine whether increased β-catenin degradation is mediated by the ubiquitin–proteasome pathway, we stimulated gastric cancer cells in the presence of the proteasome inhibitor MG132 and found that MG132 exhibited no obvious effect on Sophoridine-induced β-catenin degradation. Furthermore, expression levels of phosphorylation of GSK-3β and ubiquitin E3 ligase TRIM33 were unchanged in response to Sophoridine treatment. Ubiquitin-proteasome mediated β-catenin degradation either in GSK-3β-dependent (β-TrCP) or -independent ways (IRF2BPL and TRIM33) [31]. In the present study, we demonstrated that Sophoridine-induced β-catenin degradation was not depended on ubiquitin–proteasome pathway but depended on ESRRG, which enhances β-catenin degradation in an ubiquitin-proteasome independent manner [21]. Since ESRRG is a downstream signaling protein of MAPK pathways and the activation of MAPKs ERK1/2, p38 and JNK1/2 promote the phosphorylation of β-catenin [32–34], Sophoridine may enhance β-catenin degradation via an MAPK/ESRRG pathway.

As a member of nuclear receptors (NR) superfamily of transcription factors, ESRRG has been identified as a tumor suppressor and an attractive therapeutic target in human breast, thyroid, prostate, endometrial and gastric cancers [21, 35]. Mechanism analyses have revealed that ESRRG plays a key role in fatty acid oxidation and suppresses proliferation of both androgen-sensitive and -insensitive prostate cancer cell via the induction of p21^WAF1/CIP1^ and p27^KIP1^ [36]. In addition, ESRRG also reverses EMT process via directly inducing E-cadherin upregulation. DN200434, the recently discovered orally bioavailable agonist of ESRRG, enhanced radioiodine therapy responsiveness in thyroid cancer with either KRAS or BRAF mutations both in vitro and in vivo [37]. Thus, with the ability to enhance the expression and function of ESRRG, Sophoridine is promising to be a new and effective inducer of ESRRG.

## Conclusions

In summary, the present study demonstrated the tumor-suppressive effects and potential molecular mechanisms of Sophoridine in human gastric cancer cells. Sophoridine significantly inhibits survival, invasion and migration through enhancing ESRRG expression, which leads to the degradation of β-catenin. Moreover, Sophoridine induces G2/M cell cycle arrest via inhibiting double-stranded DNA breaks repair and enhances the efficacy of cisplatin in gastric cancer cells. Thus, as a potential anti-cancer agent, Sophoridine is promising to be a new promising therapeutic strategy for gastric cancer.

## Supplementary information

**Additional file 1: Figure S1.** (A) SGC7901 cells were treated with indicated concentrations of SOP for 24 h, PI stain and flow cytometry analysis were performed to assess cell cycle distribution. (B and C) AGS cells were treated with 5 ng/ml TGF-β alone or in combination with 3 μM SOP for 24 h, E-cadherin expression was further determined by immunofluorescence (B) and flowcytometry (C) analysis. Abbreviation: SOP, Sophoridine.

**Additional file 2: Figure S2.** SOP enhances β-catenin degradation by ESRRG in gastric cancer cells (related to Fig. 4). Band density of target proteins in Fig. 4 was quantified by ImageJ software (Version 6.0, Media Cybernetics, Inc.) and normalized to indicated control. The results are representatives of at least 3 independent experiments. Data were presented as mean ± SD. *P < 0.05; **P < 0.01; ***P < 0.0001. Abbreviation: SOP, Sophoridine.

**Additional file 3: Figure S3.** SGC7901 cells transfected with siRNA non-targeted control (siNC) or ESRRG siRNA were treated with or without 3 μM SOP for 24 h, (A) EDU assay was used to evaluate cell viability; (B) Statistical analysis of apoptotic SGC7901 cells. (C) Related to Fig. 5, PI stain and flow cytometry analysis were performed to assess cell cycle distribution in AGS cells. Same as (A), (D) PI stain and flow cytometry analysis were performed to assess cell cycle distribution in SGC7901 cells; (E) migration and invasion of SGC7901 cells were determined by transwell assay. The results are representatives of at least 3 independent experiments. Data were presented as mean ± SD. **P < 0.01; ***P < 0.0001; *NS*, no significant difference. Abbreviation: SOP, Sophoridine.

**Additional file 4: Figure S4.** Original full-length blots/gels of the western blot in Fig. 2. The cropping of the blot by figure processing software was clearly mentioned with red rectangle. Abbreviation: SOP, Sophoridine.

**Additional file 5: Figure S5.** Original full-length blots/gels of the western blot in Fig. 3. The cropping of the blot by figure processing software was clearly mentioned with red rectangle. Abbreviation: SOP, Sophoridine.

**Additional file 6: Figure S6.** Original full-length blots/gels of the western blot in Fig. 4. The cropping of the blot by figure processing software was clearly mentioned with red rectangle. Abbreviation: SOP, Sophoridine; CHX: cycloheximide.

## Data Availability

The data used to support the findings of this study are included within the article. The data and materials in the current study are available from the corresponding author on reasonable request.

## Abbreviations

ESRRG: Estrogen-related receptor gamma
TAM: Tumor-associated macrophages
siNC: siRNA non-targeted control
γH2AX: phosphorylated H2AX
EMT: Epithelial-mesenchymal transition
CHX: Cycloheximide
