# Supplementary figures and images for: Sophoridine exerts tumor-suppressive activities via promoting ESRRG-mediated β-catenin degradation in gastric cancer
Source: BMC Cancer. 2020 Jun 22;20:582. doi: 10.1186/s12885-020-07067-x (PMC7310191; doi:10.1186/s12885-020-07067-x)

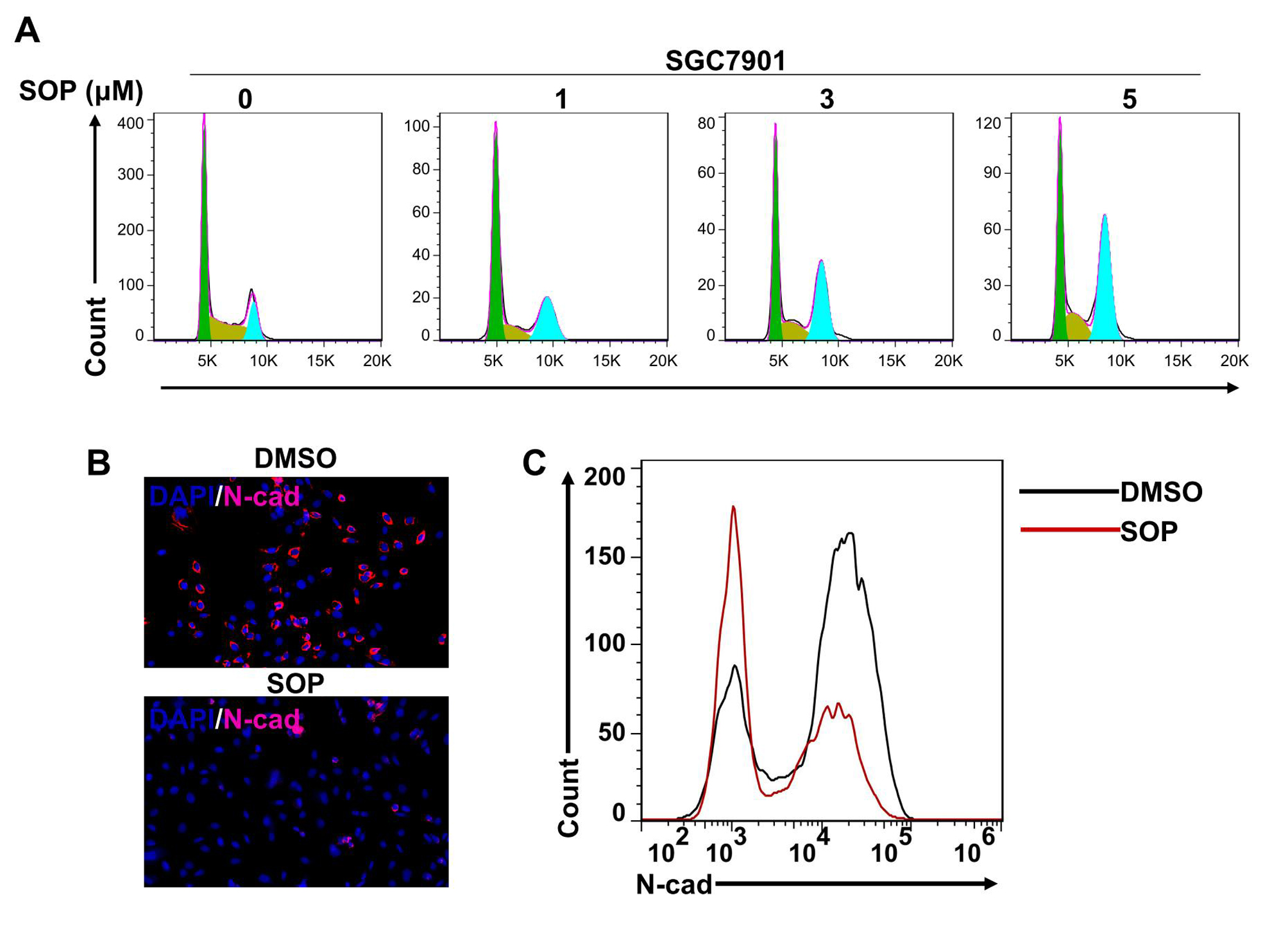

Supplement: Supplementary file 1 — Additional file 1: Figure S1. (A) SGC7901 cells were treated with indicated concentrations of SOP for 24 h, PI stain and flow cytometry analysis were performed to assess cell cycle distribution. (B and C) AGS cells were treated with 5 ng/ml TGF-β alone or in combination with 3 μM SOP for 24 h, E-cadherin expression was further determined by immunofluorescence (B) and flowcytometry (C) analysis. Abbreviation: SOP, Sophoridine. [file 12885_2020_7067_MOESM1_ESM.jpg]

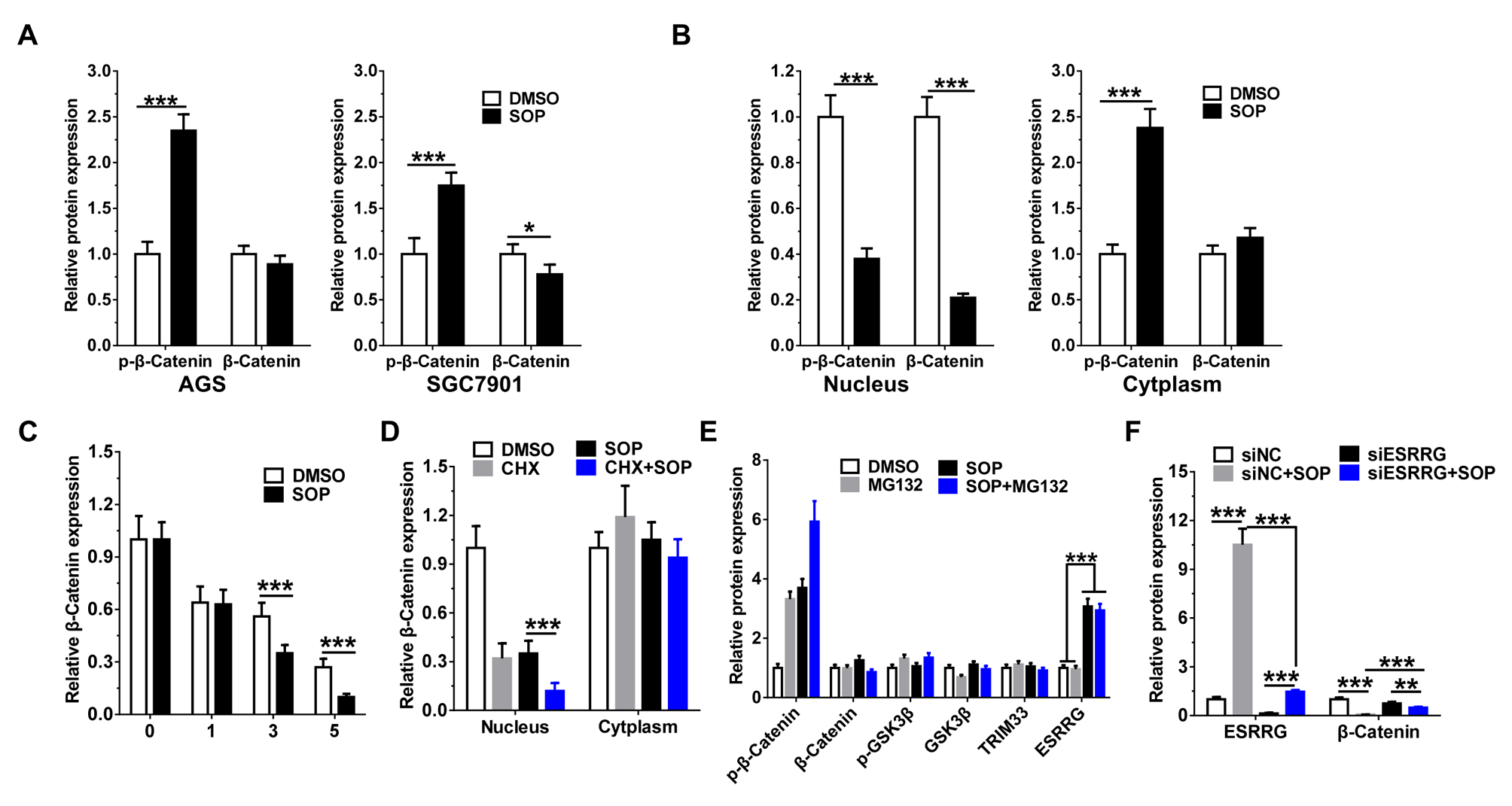

Supplement: Supplementary file 2 — Additional file 2: Figure S2. SOP enhances β-catenin degradation by ESRRG in gastric cancer cells (related to Fig. 4). Band density of target proteins in Fig. 4 was quantified by ImageJ software (Version 6.0, Media Cybernetics, Inc.) and normalized to indicated control. The results are representatives of at least 3 independent experiments. Data were presented as mean ± SD. *P < 0.05; **P < 0.01; ***P < 0.0001. Abbreviation: SOP, Sophoridine. [file 12885_2020_7067_MOESM2_ESM.tif]

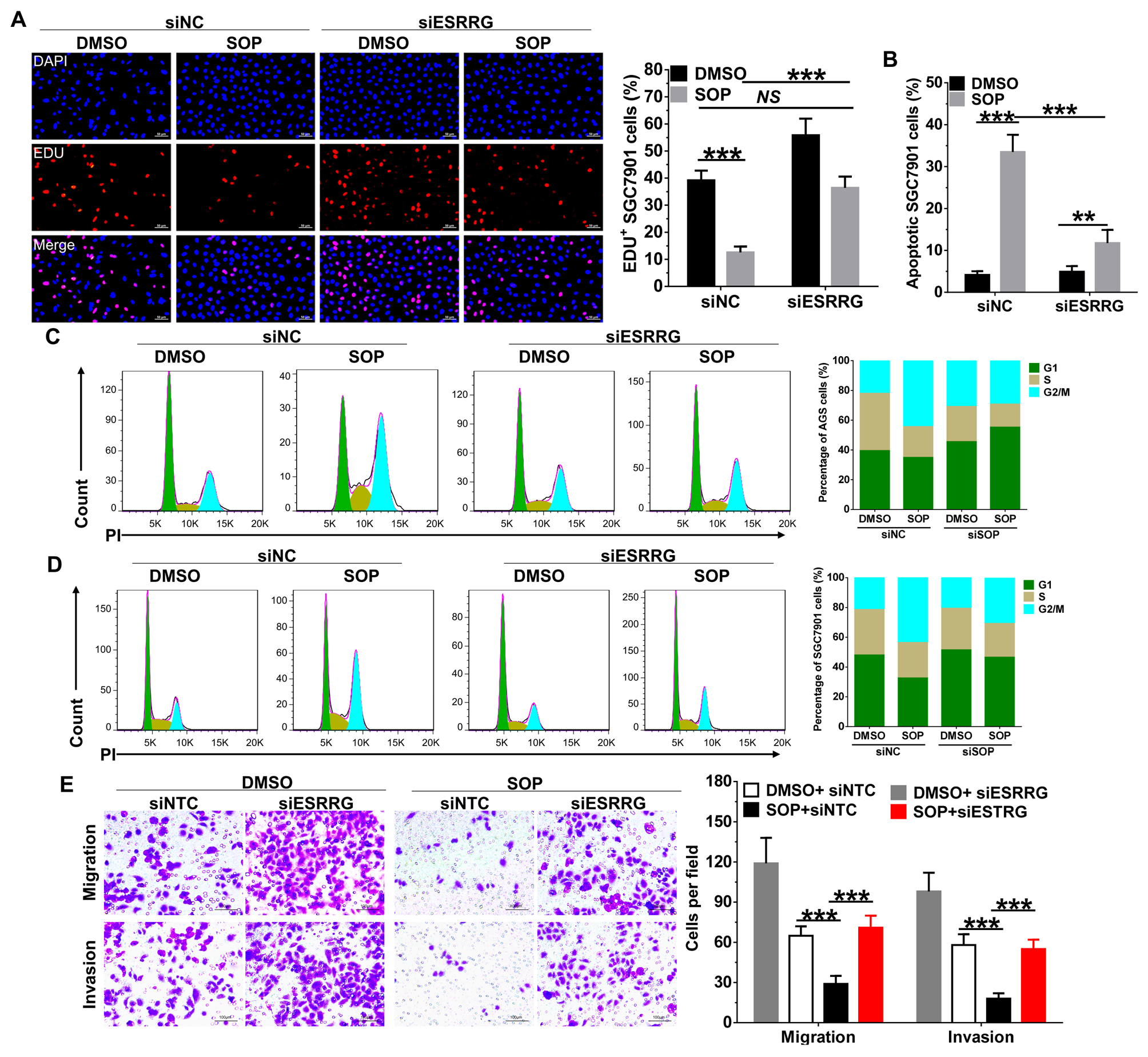

Supplement: Supplementary file 3 — Additional file 3: Figure S3. SGC7901 cells transfected with siRNA non-targeted control (siNC) or ESRRG siRNA were treated with or without 3 μM SOP for 24 h, (A) EDU assay was used to evaluate cell viability; (B) Statistical analysis of apoptotic SGC7901 cells. (C) Related to Fig. 5, PI stain and flow cytometry analysis were performed to assess cell cycle distribution in AGS cells. Same as (A), (D) PI stain and flow cytometry analysis were performed to assess cell cycle distribution in SGC7901 cells; (E) migration and invasion of SGC7901 cells were determined by transwell assay. The results are representatives of at least 3 independent experiments. Data were presented as mean ± SD. **P < 0.01; ***P < 0.0001; NS, no significant difference. Abbreviation: SOP, Sophoridine. [file 12885_2020_7067_MOESM3_ESM.tif]

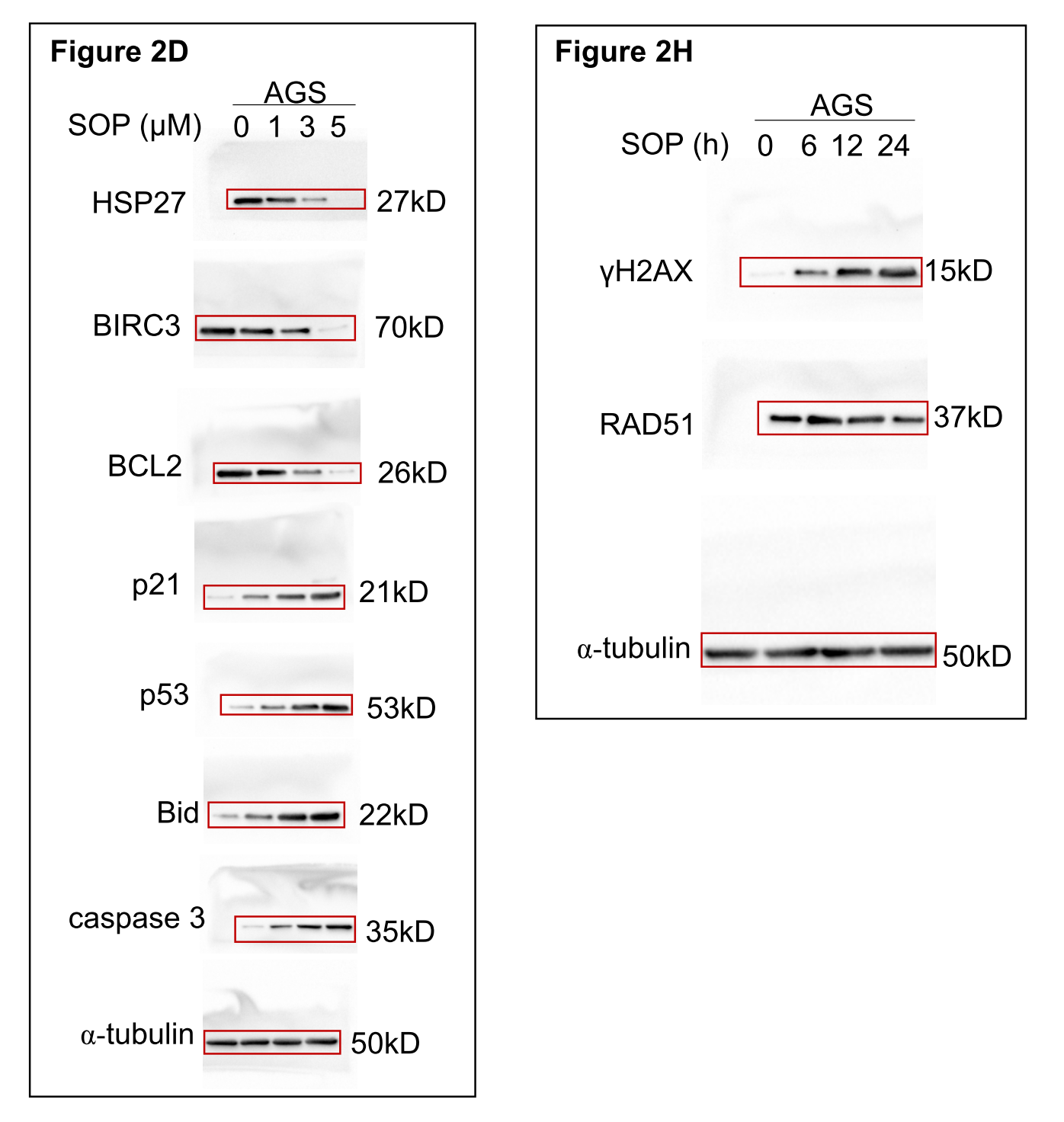

Supplement: Supplementary file 4 — Additional file 4: Figure S4. Original full-length blots/gels of the western blot in Fig. 2. The cropping of the blot by figure processing software was clearly mentioned with red rectangle. Abbreviation: SOP, Sophoridine. [file 12885_2020_7067_MOESM4_ESM.tif]

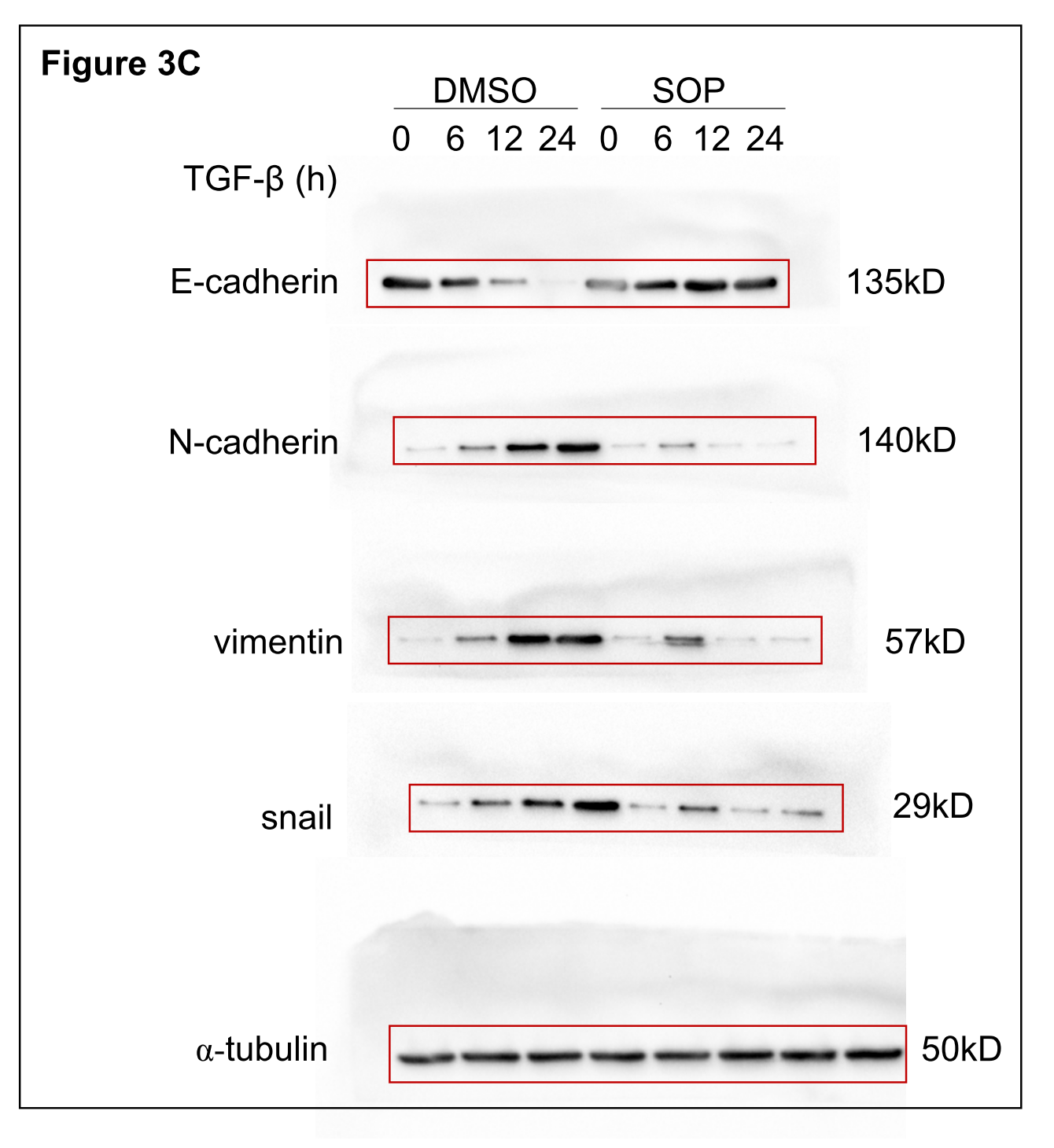

Supplement: Supplementary file 5 — Additional file 5: Figure S5. Original full-length blots/gels of the western blot in Fig. 3. The cropping of the blot by figure processing software was clearly mentioned with red rectangle. Abbreviation: SOP, Sophoridine. [file 12885_2020_7067_MOESM5_ESM.tif]

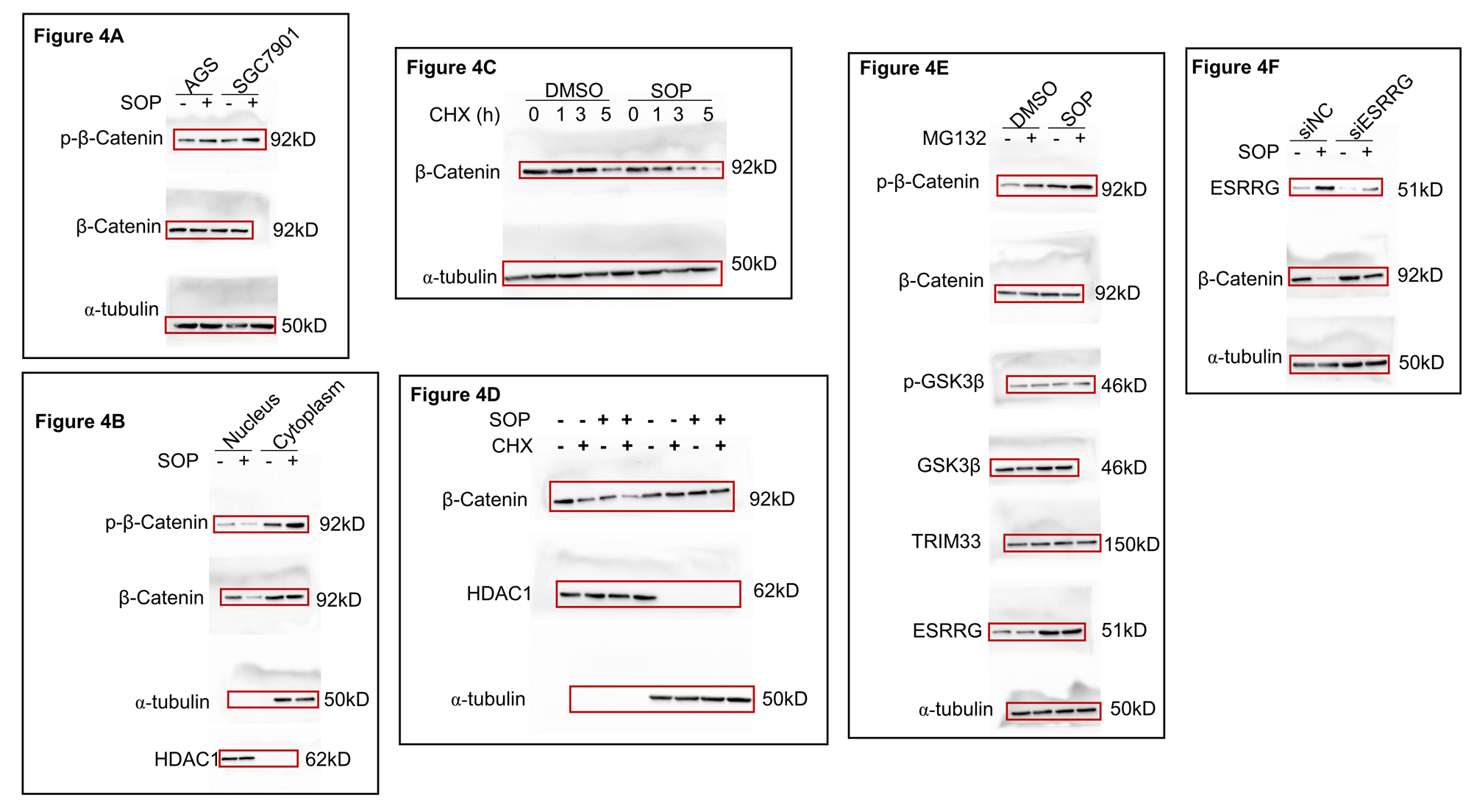

Supplement: Supplementary file 6 — Additional file 6: Figure S6. Original full-length blots/gels of the western blot in Fig. 4. The cropping of the blot by figure processing software was clearly mentioned with red rectangle. Abbreviation: SOP, Sophoridine; CHX: cycloheximide. [file 12885_2020_7067_MOESM6_ESM.tif]
